# Bodyweight, locomotion, and behavioral responses of the naked mole rat (*Heterocephalus glaber*) to lipopolysaccharide administration

**DOI:** 10.1007/s00359-022-01557-y

**Published:** 2022-06-22

**Authors:** Mosiany Letura Kisipan, Rodi Omondi Ojoo, Titus Ikusya Kanui, Klas S. P. Abelson

**Affiliations:** 1 Department of Experimental Medicine, University of Copenhagen, Copenhagen, Denmark; 2 Department of Veterinary Anatomy and Physiology, Egerton University, Njoro, Kenya; 3 Department of Veterinary Anatomy and Physiology, University of Nairobi, Nairobi, Kenya; 4 Department of Agricultural Sciences, South Eastern Kenya University, Kitui, Kenya

**Keywords:** Naked mole rat, Bodyweight, Behavior, Locomotor activity, Open field test

## Abstract

The naked mole rat has unique biologic characteristics that include atypical inflammatory responses. Lipopolysaccharide induces inflammation which triggers brain centers controlling feeding, and behavior to result in “sick animal behavior”. We characterized the bodyweight, locomotor, and other behavioral responses of this rodent to lipopolysaccharide administration. Lipopolysaccharide caused weight losses, which were not prevented by TAK 242. In the open field test, lipopolysaccharide did not depress locomotion, while urination, defecation, and activity freezing were rare. The animals exhibited walling but not rearing and fast backward movements that were unaffected by lipopolysaccharide. Failure to depress locomotion suggests either a unique immunity-brain crosstalk or motor responses/centers that tolerate depressive effects of inflammation. The absence of activity freezing and rarity of urination and defecation suggests that novel environments or lipopolysaccharide do not induce anxiety, or that anxiety is expressed differently in the animal. The absence of rearing could be due to the design of the animal’s locomotor apparatus while fast backward movement could be a mechanism for quick escape from threats in the tunnels of their habitat. Our results elucidate the unique biology of this rodent, which elicits interest in the animal as a model for inflammatory research, although the findings require mechanistic corroborations.

**Supplementary Information:**

The online version contains supplementary material available at 10.1007/s00359-022-01557-y.

## Introduction

The naked mole rat (NMR, *Heterocephalus glaber*) is a subterranean mouse-sized eusocial rodent distributed in the eastern horn of Africa (Jarvis 1981; Bourke 1991). The animal has several unique biologic characteristics. These include cancer resistance, tolerance to hypoxia, pain, and itch; and an extended lifespan and health span compared to other rodents despite high oxidative damage (Andziak et al. 2006; Buffenstein 2008; Larson and Park 2009; Liang et al. 2010; Smith et al. 2010; Tian et al. 2015; Omerbašić et al. 2016; Tan et al. 2017; Kurz et al. 2017; Ruby et al. 2018). The animal has also been shown to exhibit resistance to age-related and inflammatory conditions (Hilton et al. 2019; Kisipan et al. 2020; Taguchi et al. 2020). Due to its unique biological attributes, the animal has been found of great interest in biomedical research.

Lipopolysaccharide (endotoxin, LPS) is a major component of the outer membrane of gram-negative bacteria, which is detected by toll-like receptor 4 (TLR4). Activated TLR4 in turn induces transcription and release of cytokines and chemokines that trigger the host’s innate immune response and inflammation geared toward the elimination of the pathogens (Beutler 2002; Vaure and Liu 2014; Rosadini and Kagan 2017; Kuzmich et al. 2017). The inflammatory responses to LPS are accompanied by loss of body weight and physiological and behavioral changes referred to as “sick animal behavior” characterized by anhedonia, lethargy, loss of appetite, anxiety, and sleepiness (Engeland et al. 2001; Bassi et al. 2012; Piirsalu et al. 2020; Vichaya et al. 2020; Lasselin et al. 2020). Administration of this agent is therefore commonly used to model sepsis, neuroinflammation, and organ injuries, as well as behavioral and other aspects of sickness in laboratory animals (Bassi et al. 2012; Seemann et al. 2017; Catorce and Gevorkian 2020; Lasselin et al. 2020).

TAK 242 is a small, synthetic molecule derived from cyclohexene (Yamada et al. 2005; Ii et al. 2006). This molecule is a specific inhibitor of TLR4, binding to the intracellular domain of the receptor (Ii et al. 2006; Kawamoto et al. 2008; Matsunaga et al. 2011; Ono et al. 2020). Through its action, TAK 242 inhibits LPS-induced cytokine production and inflammation (Ono et al. 2020; Samarpita et al. 2020). In preclinical models, TAK 242 has been shown to protect against inflammation-induced organ injuries (Fenhammar et al. 2011; Zhang et al. 2015; Bhattacharyya et al. 2018) and when used with an antibiotic, the agent improved survival in a mouse sepsis model (Takashima et al. 2009). Some studies have also reported that the molecule prevents endotoxemia-induced muscle wasting (Ono et al. 2020), motor dysfunctions (Fellner et al. 2017), and some behavioral changes due to inflammation or stress (Zhang et al. 2020).

The immune system and inflammation play a key role in both aging and cancer (Coussens and Werb 2002; Ferrucci and Fabbri 2018; Neves and Sousa-Victor 2020; Zhao et al. 2021). In NMR, the immune system exhibits some peculiarities in terms of cellular composition with a predominance of myeloid lineage cells and a lack of natural killer cells (Cheng et al. 2017; Hilton et al. 2019). It is therefore likely that the immune system of the NMR is a key contributor to its prolonged health span and cancer resistance and the same has been speculated in recent reports (Zhao et al. 2014; Hilton et al. 2019; Kisipan et al. 2020; Taguchi et al. 2020). The establishment of this animal as a model for aging, cancer, and inflammatory diseases, therefore, requires characterization of the immunity and its interactions with the other body systems and biological processes in the animal.

The alterations in behavior and spontaneous locomotor activity of animals due to LPS are mediated by signals from the immune system which have been demonstrated to alter the activity of brain centers that regulate or control behavior and activity (Kim et al. 2013; Piirsalu et al. 2020). The aforementioned unique immunosurveillance of the NMR could confer the animal an atypical sick animal behavior phenotype in response to activation of the immune system. The present study, therefore, aimed to characterize body weight, locomotor responses as well as other behavioral responses of NMR to systemic administration of LPS. The study also aimed to test whether TAK 242 would alleviate body weight loss as well as behavioral/locomotor changes that may occur in response to inflammation elicited by LPS.

## Materials and methods

### Animals

NMRs were captured from Kibwezi in Makueni County, Kenya, and kept in a dark temperature-controlled room (maintained at 28–31 °C with 50–70% relative humidity) at the main campus of the South Eastern Kenya University. The animals were randomly distributed into seven groups of 5–14 animals (see details under the “study design” subsection below), each housed in an acrylic glass cage constructed following a design in a recent study on the housing behavior of NMR (Mwobobia et al. 2020). The cages measured 70 cm, 50 cm, and 20 cm respectively for length, width, and height and were partitioned into three interconnected compartments with wood shavings as bedding material. The animals had an acclimatization period of four months and were fed fresh sweet potatoes and carrots. The amount of feed during acclimatization was approximately 10% of the total weight of the animals in a particular group and was supplied between 8.00 and 10.00 am. A week before the start of the experiment, the feeding regime was changed to ad libitum. Water was not provided, since animals do not drink in their natural habitat as they receive sufficient fluid via the feed (Park et al. 2009).

### Chemicals

LPS (E. coli O111:B4) was purchased from Sigma-Aldrich (catalog No. L2630-25MG). This was dissolved in normal saline to make a stock solution of 5 mg/ml from which an aliquot was taken and diluted to a working solution of 0.25 mg/ml and 0.125 mg/ml. TAK 242 was purchased from Tocris bioscience (Catalog No. 6587). TAK 242 was first dissolved in ethanol and then further diluted with normal saline to a final concentration of 0.25 mg/ml, with an ethanol concentration of 2.5%.

### Locomotor activity

Locomotor activity (LA) was analyzed in an open field test (OFT) observation chamber made of acrylic glass, measuring 48 cm, 48 cm, and 20 cm in length, width, and height, respectively. The chamber had 12 cm square grid lines inscribed on its base/floor to give a total of 16 squares. An ordinary video camera was mounted over the chamber to record the activity from an aerial view. To analyze LA, the video camera was turned on and an individual animal was gently placed at the center of OFT chamber. The recording lasted 5 min, after which the animal was placed back in its cage. Between observations of each animal, the cage was wiped with 80% ethanol and then allowed to dry for 3 min before the next animal was placed in the chamber.

### Study design

A preliminary experiment to record the basal locomotor activity of untreated NMRs was conducted using one group of five animals. The other animals were randomly assigned to six groups of 8–14 individuals each, which received an intraperitoneal injection of either saline (1 ml) or an amount of test agents in milligrams (mg) per kilogram (kg) body weight (bwt). The sample size was based on literature from similar studies using laboratory rodents (Lawrence et al. 2012; Kim et al. 2013; Sulakhiya et al. 2016; Zhong et al. 2018) and calculations using experimental design assistant (EDA) web application (NC3Rs 2022; Percie du Sert et al. 2017) with power set at 80% and significance level of 0.05. For bodyweight analysis, the effect size was set at 10, and the standard deviation of 6 while in LA and behavioral analyses, the effect size was set at 100 and the standard deviation at 50. No animal was injected with a volume exceeding 1 ml. The agents were administered either at the start of the experiment or at 3 h as summarized in Table 1. Bodyweight of each animal was recorded at the start of the experiment and once daily for the next 4 days. LA was analyzed in the observation chamber 6 h after the start of the experiment (Day 1) and then on the following day (Day 2). Due to a technical hitch, LA was not recorded on Day 1 for TAK 242 pre-treatment groups. but were instead recorded Day 2 and 3.

**Table 1:**
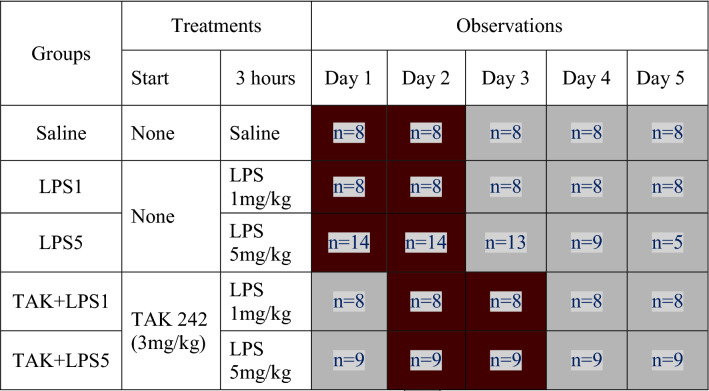
A summary of the study design showing the test agents, their schedule of administration and analyses done in the NMR

Body weight and LA/behavioral analysis,  Body weight alone

### Data analysis

Bodyweight data were compared using two-way ANOVA and this was followed by post-hoc Bonferroni test to compare data for different treatments on a particular day of the experiment. Bodyweight change was calculated as a proportion of body weight gained or lost either between two consecutive days (for daily weight change) or between the first and the last day of the experiment (for overall weight change). The calculations were done as follows:

$${\text{Daily}}\,{\text{weight}}\,{\text{change}}\, = \,({\text{wtb}} - {\text{ wta}})/{\text{wta}}$$

$${\text{Overall}}\,{\text{ weight}}\,{\text{ change}}\, = \,({\text{wt5}} - {\text{ wt1}})/{\text{wt1}}$$

where:

wta is the body weight recorded on the day preceding that of the weight change recording.

wtb is the body weight on a particular day of weight change recording.

wt1 is the bodyweight recorded at the start of the experiment (on Day 1)

wt5 is the body weight recorded at the end of the experiment (on Day 5)

Bodyweight change data for different groups were compared using two-way ANOVA followed by post-hoc Bonferroni test.

The video clips that captured LA were viewed and the locomotor activity of the animal was analyzed. The number of line crossings, walling/rearing, grooming bouts, defecations, urinations as well as other motor or behavioral activities were counted and recorded for each animal by a blinded examiner with the aid of a tally counter. Line crossings, wallings/rearings, and grooming bouts were compared between the groups using two-way ANOVA followed by post-hoc Bonferroni test. The total number of fecal boluses and falls were recorded. In all statistical analyses, *p* < 0.05 was considered statistically significant.

## Results

### General observations

Animals injected with LPS, either alone or after pre-treatment with TAK 242, showed marked dullness, reduced movement, and lack of appetite. These signs were extreme in two animals in the LPS5 group, one each on Day 3 and Day 4. These animals were considered to have reached a pre-determined humane endpoint and thus euthanized on welfare grounds. No data from these two animals were included in the analysis.

### Bodyweight

**Fig. 1:**
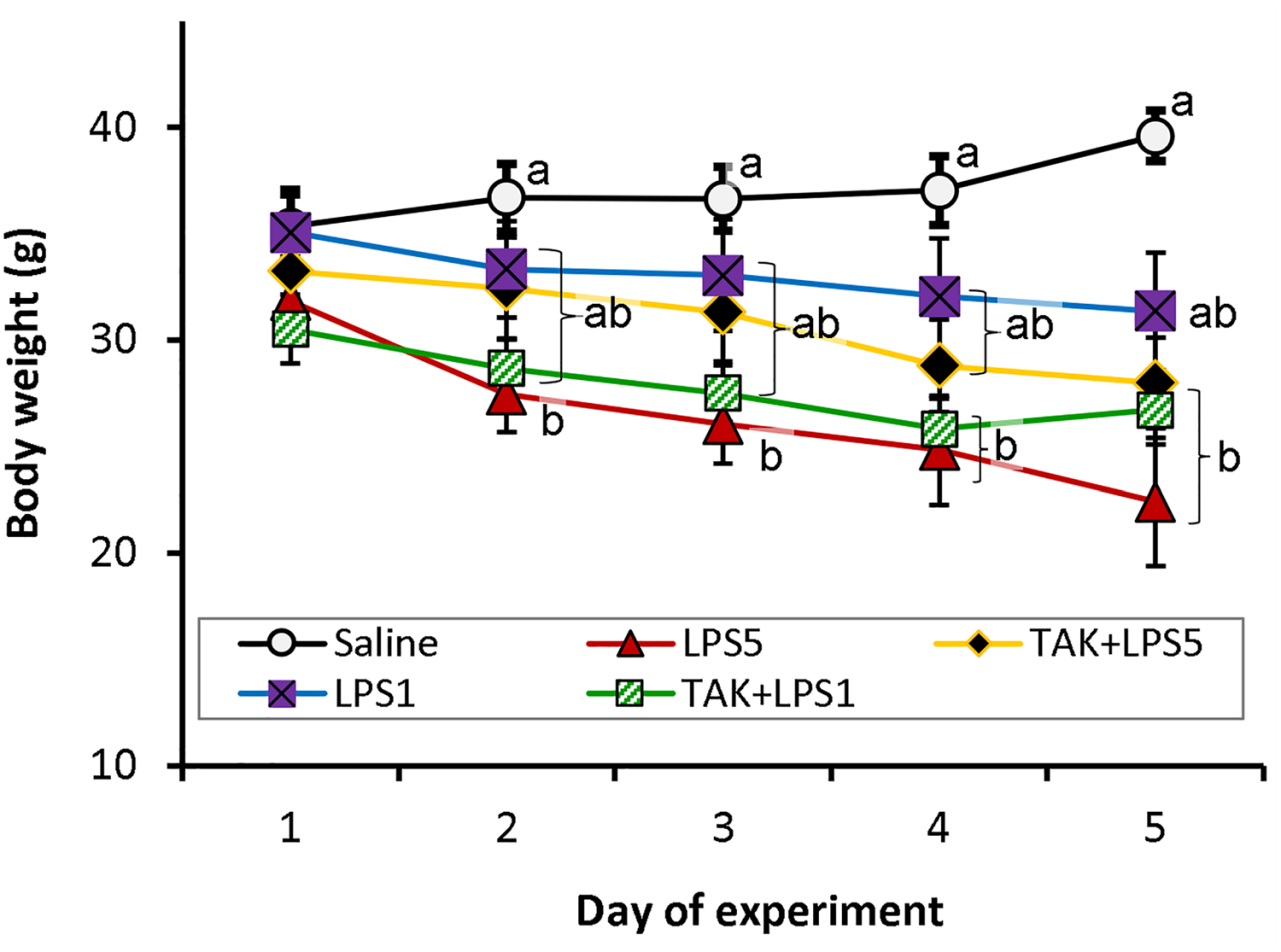
NMR body weight values at different days of the study. Different letters indicate differences between treatments within a particular day of experiment. Data are expressed as mean $\pm$ SEM

All bodyweight data recorded over the entire period of the experiment (a total of 217 observations) showed significant variations between treatment groups (ANOVA: *F*_4, 212_ = 18.31, *p* < 0.001) while the variation between days was marginal (ANOVA: *F*_4, 212_ = 2.42, *p* = 0.05) with no interaction between the two factors (*p* = 0.78). On Day 1 of the experiment, the mean weights for all the groups did not differ significantly (ANOVA: *F*_4,42_ = 0.92, *p* > 0.46, Fig. 1). In each of the subsequent days, there were significant differences between at least two groups (*p* $\le 0.01,$ see Table 2). All the treated groups had lower body weights compared to the controls with the difference being significant in LPS5 throughout the study (*p* $\le$ 0.01), TAK + LPS1 on Days 3,4, and 5 (*p* ≤ 0.05), and TAK + LPS5 on Day 5 (*p* = 0.018).

**Table 2:** A summary of ANOVA testing the difference in body weight between groups at different days of experiment

	Source of variation	*df*	*F*	Prob > *F*
Day 1	Between groups	4	0.92	0.46
	Within groups	42		
Day 2	Between groups	4	3.7	0.01*
	Within groups	42		
Day 3	Between groups	4	4.53	0.004*
	Within groups	41		
Day 4	Between groups	4	5.07	0.002*
	Within groups	37		
Day 5	Between groups	4	5.89	0.001*
	Within groups	30		

The asterisk (*) indicate there is a significant difference in body weight between at least two groups

**Fig. 2:**
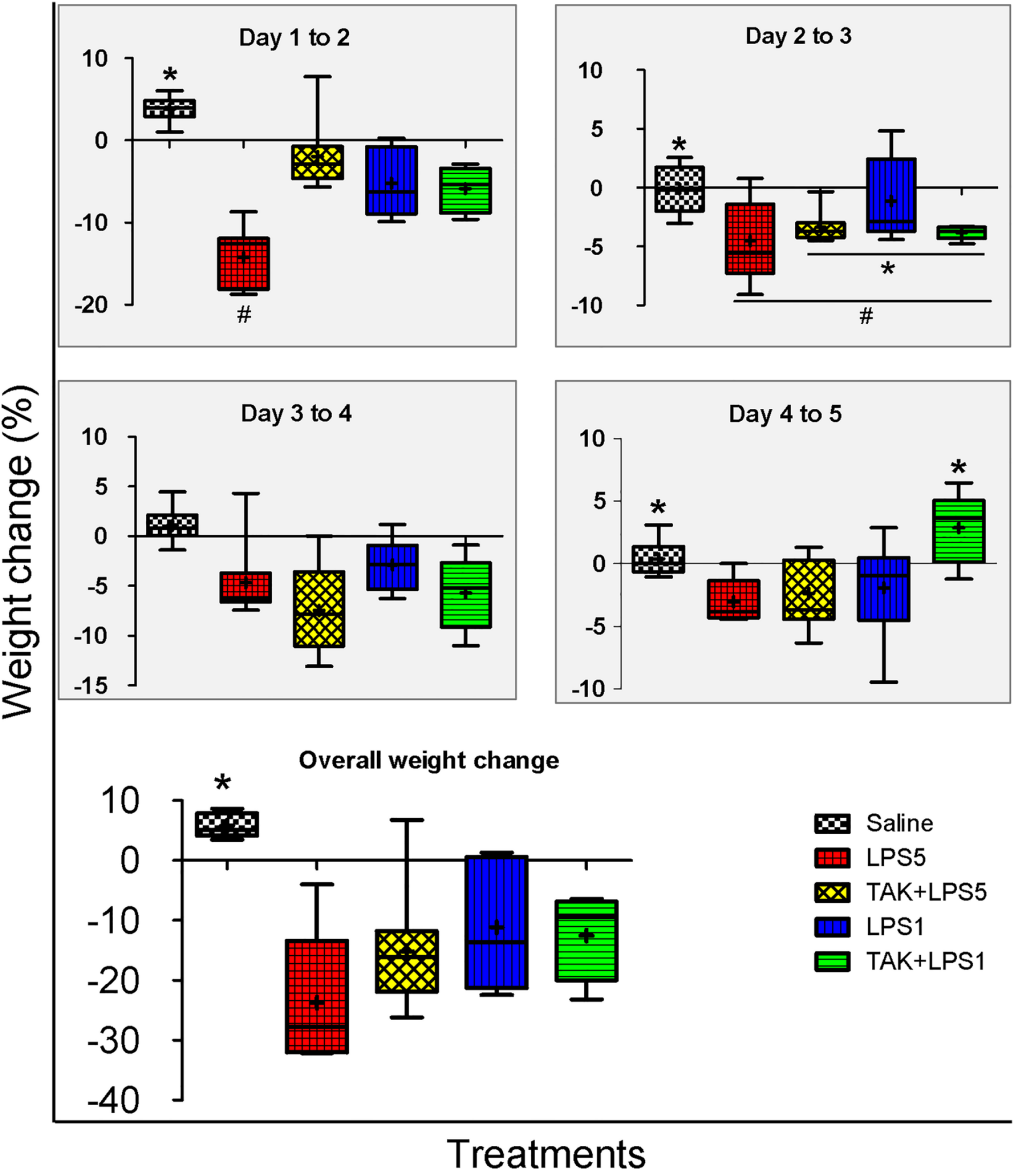
Box and whisker plots showing changes in body weight of NMRs injected with saline or different regimes of LPS. Control animals showed an increase in body weight while those treated with different regimes of LPS generally showed a fall in body weight. The overall weight loss did not differ significantly between groups treated with different regimes of LPS

Daily changes in mean body weight (from a grand total of 170 recordings in the entire study) varied between groups (ANOVA: *F*_(4, 166)_ = 9.2, *p* < 0.001) and day of weight change recording (ANOVA: *F*_(3, 167)_ = 4.3, *p* = 0.01, Fig. 2). Generally, saline-injected controls showed a slight increase in body weight while LPS-treated animals showed a reduction (Figs. 1 and 2). Between days 1 and 2, saline-treated controls had a slight gain in mean weight (3.8 ± 0.54%) while the changes in the subsequent days were negligible (Fig. 2). All the treated groups, on the other hand, exhibited weight losses throughout the study period except the TAK + LPS1 group which showed a slight weight gain on Days 4 to 5 period. In all daily intervals, there were significant differences in weight change between at least two groups except on Days 3 to 4 period (see Table 3). Between Day 1 and Day 2 of the study, animals injected with LPS 5 mg/kg bwt showed the most severe loss of body weight (14.3 ± 0.91%) which was statistically significant as compared to all the other groups (*p* ≤ 0.001). The differences between the weight losses in the groups that received the other regimes of LPS treatment were not statistically significant (*p* ≥ 0.2) in this period. In Day 2–3 and Day 3–4 periods, bodyweight losses were not significantly different among the LPS-treated groups (*p* ≥ 0.13). In the Day 4–5 period, the TAK + LPS1 group had a slight gain in body weight which was generally similar to that seen in controls (*p* > 0.9) while the differences between the other LPS-treated groups were not significant (*p* > 0.9).

**Table 3:** A summary of ANOVA testing the difference in body weight changes between groups in different periods of experiment

	Source	*df*	*F*	Prob > *F*
Day 1–2	Between groups	4	42.8	0.001*
	Within groups	42		
Day 2–3	Between groups	4	5.0	0.002*
	Within groups	41		
Day 3–4	Between groups	4	1.3	0.3
	Within groups	38		
Day 4–5	Between groups	4	5.0	0.003*
	Within groups	30		

The asterisk (*) indicate there is a significant difference in weight change between at least two groups

The overall change in body weight differed significantly between groups (ANOVA: *F*_(4,30)_ = 7.44, *p* ≤ 0.02). Control animals, with an overall weight gain of 5.8 ± 0.72%, had a significantly different overall weight change compared to all those treated with different regimes of LPS (*p* ≤ 0.02, Fig. 2). Animals injected with LPS 5 mg/kg bwt, showed the most severe overall weight loss but the differences between all the groups treated with LPS were not statistically significant (*p* ≥ 0.19).

### Locomotor activity

In their home cages, NMRs generally crowded together usually lying on one another to form a “heap of animals” when resting. When moving about in the cage, it was not uncommon to observe NMRs moving backwards, sometimes at relatively high speed especially when they encounter an obstacle or when “escaping” from being handled. The same was also observed in untreated and saline-injected controls. Animals injected with LPS, either alone or after pre-treatment with TAK 242, showed a marked reduction in movement, shivering, staggering gait, and loss of tendency to heap on one another.

**Fig. 3:**
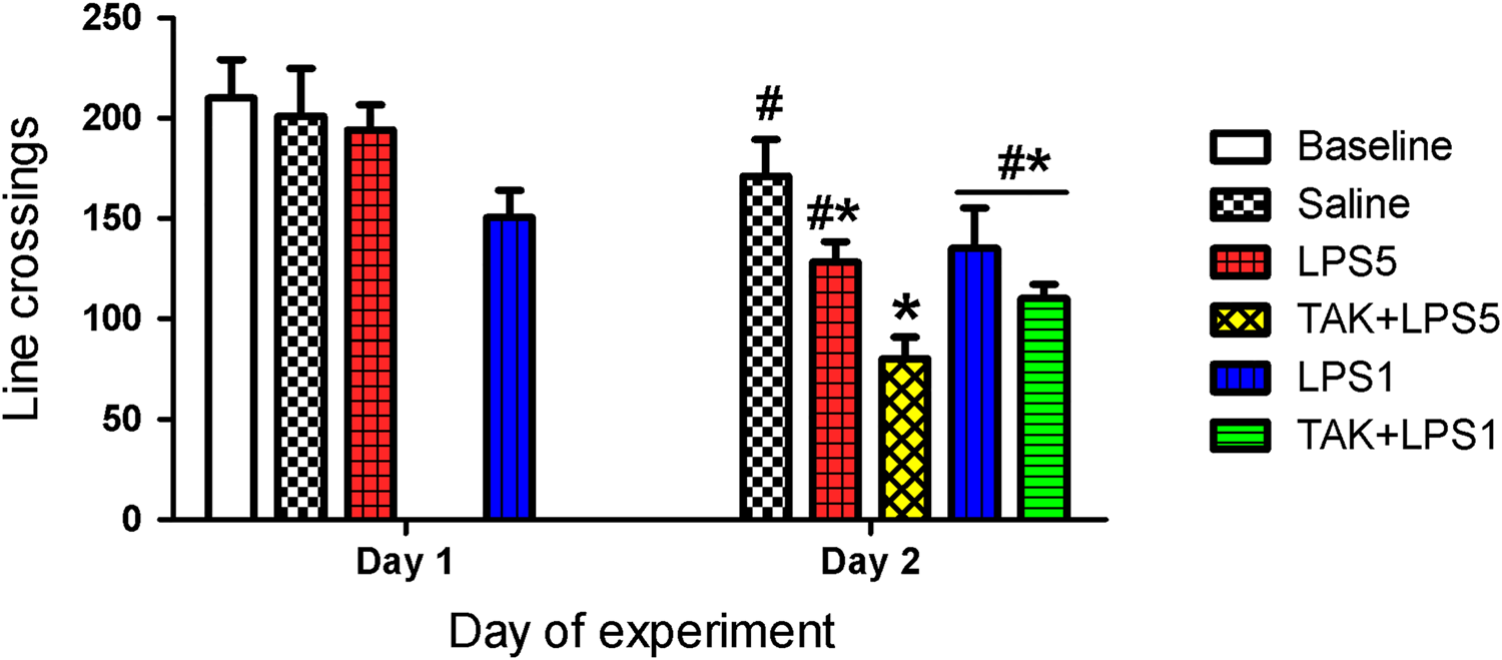
Spontaneous locomotor activity (LA) of NMRs quantified as number of line crossings. LA generally did not differ significantly between both the untreated and saline-injected controls and LPS-injected animals except for significantly lower values for TAK + LPS5 group as compared to controls in Day 2

In the OFT chamber, NMRs exhibited continuous movement that started immediately after placement in the chamber and lasted until the end of activity recording. Sniffing and stretched-attend posture was not exhibited but the animals rather moved about in the chamber without the “typical” investigative behaviors seen in rodents. The basal spontaneous locomotor activity (LA) of untreated NMRs was 210 ± 19.2 line crossings in 5 min. On Day 1 of the experiment (3 h after injection of LPS or saline), the number of line crossings recorded in the groups analyzed did not significantly differ and were generally similar to that observed in the untreated animals (ANOVA: *F*_(3,30)_ = 2.1, *p* = 0.12, Fig. 3). On Day 2, significant differences in the number of line crossings were observed (ANOVA: *F*_(4, 29)_ = 5.9, *p* = 0.001) with the values being significantly lower in TAK + LPS5 as compared to the controls (*p* = 0.001). Values for the same group were also lower as compared to LPS1 but the statistical significance was marginal (p = 0.05).

**Fig. 4:**
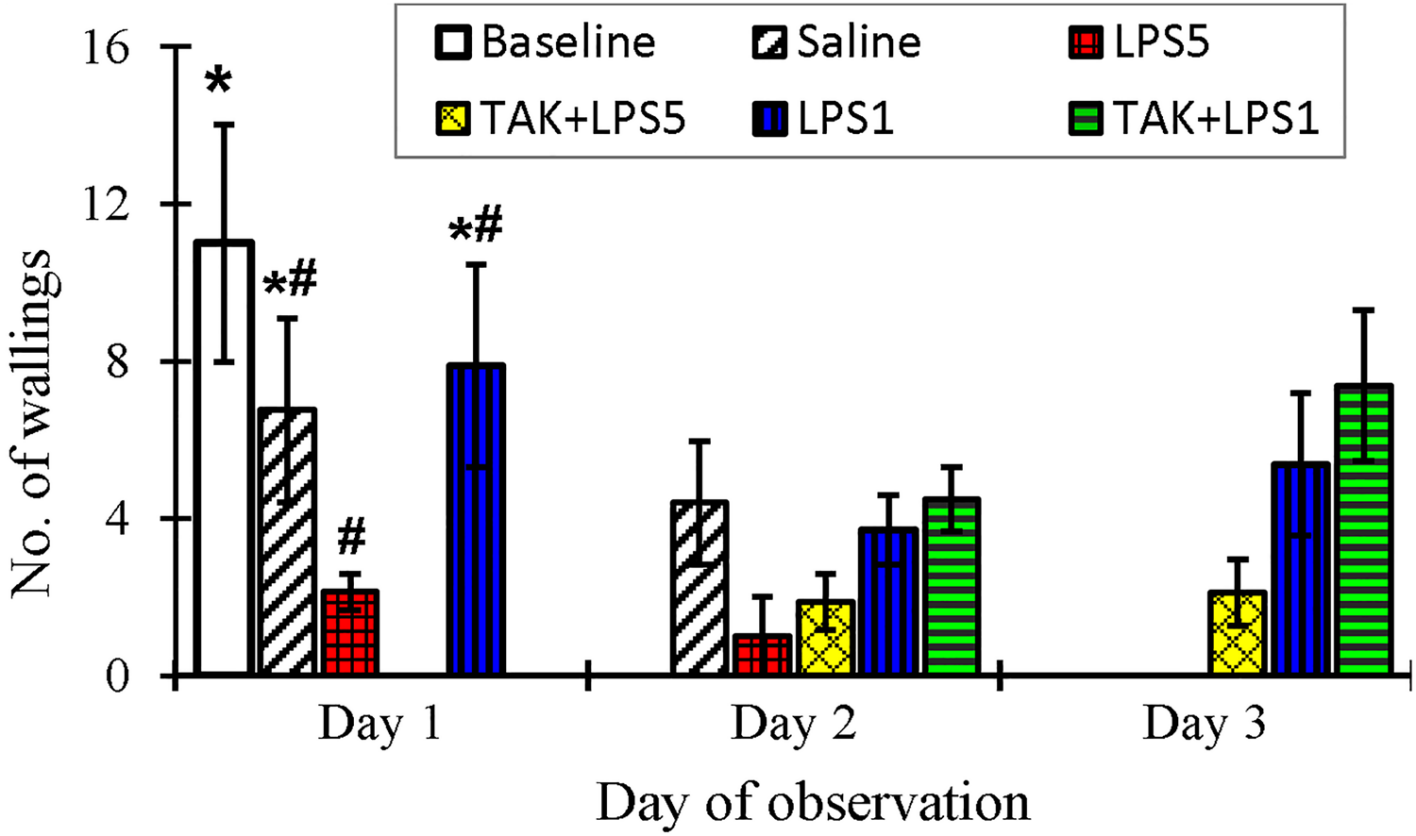
Completed walling attempts in NMR. LPS5 had the lowest number of wallings recorded in day 1 as compared to controls. Differences in groups recorded on days 2 and 3 were not significant. Data are presented as mean ± SEM

Occasional rapid backward movements were also observed in the OFT (Supplementary file 1). The occurrence and the number of backward movements (Table 4) appeared to be random with no pattern between the treatments and days of LA analysis. Rearing (standing on hindlimbs without support) was not observed but the animals could stand on hindlimbs with forepaws placed on the cage wall for support, a feature referred to as walling (Mesembe et al. 2012). The number of completed walling bouts did not significantly differ between all the treatment groups at any recording time point, except on day 1 (ANOVA: *F*_(3, 31)_ = 4.3, *p* = 0.01), where walling bouts in the untreated controls were significantly higher than in LPS5 group (Fig. 4, *p* = 0.03). In the subsequent days, the differences between the groups were not significant (*p* ≥ 0.06).

**Table 4:** Total number of lines crossed while moving backwards, grooming bouts and falls by the NMR (controls and different regimes of LPS treatment) as recorded in the OFT chamber

	Backward line crossings			Self-grooming bouts			Falls		
	Day 1	Day 2	Day 3	Day 1	Day 2	Day 3	Day 1	Day 2	Day 3
Baseline	0			3					
Saline	1	5	7	1	3	5	0	0	0
LPS5	6	0	0	6	7	7	32	0	0
TAK + LPS5	N/A	13	17	N/A	38	42	N/A	3	3
LPS1	9	23	4	9	10	42	0	0	0
TAK + LPS1	N/A	4	3	N/A	22	26	N/A	0	0

Self-grooming observed in NMR only included licking the forepaws and then rubbing the face, mainly the snout, with them. Self-grooming of the rest of the body (besides the face) was not observed. The number of grooming bouts showed significant differences between the groups on both Day 1 and Day 2 (Table 5) where the LPS1 group recorded a higher number of grooming bouts than controls on Day 1, (*p* = 0.02) while on Day 2, the bouts were higher in TAK + LPS5 compared to both the controls and LPS1 (*p* ≤ 0.03).

**Table 5:** A summary of ANOVA testing grooming bouts of NMR recorded in OFT

	Source	*df*	*F*	Prob > *F*
Day 1	Between groups	2	4.6	0.02*
	Within groups	28		
Day 2	Between groups	4	4.9	0.004*
	Within groups	29		
Day 3	Between groups	4	2.9	0.05
	Within groups	34		

The asterisk (*) indicate there is a significant difference in in mean number of grooming bouts between at least two groups

NMRs treated with LPS exhibited staggering gait with falls recorded in those treated with LPS 5 mg/kg bwt (alone or after pre-treatment with TAK 242). The total number of falls was 32, recorded on Day 1 for LPS5, and 3 falls each on Days 2 and 3 in TAK + LPS5. Defecation and urination were very rare in NMR subjected to OFT in all treatments and controls and so were the bouts of activity ‘freezing’ thus no data on these features are presented.

## Discussion

Infections lead to anorexia, weight loss, and a set of physiological and behavioral changes, collectively referred to as “sick animal behavior”. Typical changes are reduced locomotion, loss of appetite, reduced social and environmental interaction, and diminished grooming (Kinoshita et al. 2009; Vichaya et al. 2019; Lasselin et al. 2020). The invading pathogens express structural motifs, referred to as pathogen-associated molecular patterns (PAMPS), which trigger an immune response in the host through TLRs (Lu et al. 2008; Vaure and Liu 2014; Rosadini and Kagan 2017). Activated TLRs induce the production of pro-inflammatory cytokines which are relayed to the brain through neural and humoral pathways to produce the said physiological and behavioral changes (Bret-Dibat et al. 1995; Gaykema et al. 1995; Goehler et al. 1999; Konsman et al. 2002; Dantzer 2018). This illustrates part of the crosstalk or interaction between the immune and nervous systems. NMR is finding preference as a model for biomedical research due to its prolonged lifespan and resistance to age-related conditions including cancer (Buffenstein 2008; Liang et al. 2010; Tian et al. 2015). This attribute has been suggested to stem from a unique immunosurveillance mechanism of the animal. Thus, it is important that all and related aspects of the immune response are understood to establish the suitability and relevance of the animal as a model.

LPS specifically activates TLR4, which in turn induces systemic inflammation and even sepsis if the signal is excessive (Lu et al. 2008). In the present study, treatment with LPS, with or without TAK-242 pre-treatment, resulted in a significant reduction in body weight, a feature that was not seen in saline-injected controls. In our study, only two doses of LPS were used, a high dose of 5 mg/kg bwt and a low dose of 1 mg/kg bwt. The overall proportion of body weight loss was higher in animals that received a high dose, pointing to a dose-dependent response although the difference between the doses was not statistically significant. Weight loss in response to LPS has been reported to result from reduced feed intake which is in turn caused by the inflammation elicited by this agent (Kim et al. 2013; Piirsalu et al. 2020). Severely reduced feed intake was also a significant observation in LPS-injected NMRs in the present study. LPS stimulates the production of proinflammatory cytokines which are relayed to the brain to act on the hypothalamus to cause sickness behavior characterized by among others, anorexia and weight loss (Konsman et al. 2002; Kim et al. 2013). It has also been suggested that reduced feed intake after LPS administration results from activation of hypothalamic herpes virus entry mediator (HVEM) by the inflammatory cytokines (Kim et al. 2013). This aspect was however not been investigated in our study.

The open field test is commonly used to analyze behavior particularly spontaneous locomotor activity in animal models (Tatem et al. 2014; Seibenhener and Wooten 2015). LPS administration causes lethargy, anhedonia, sleepiness, and depressed locomotion (Engeland et al. 2001; Bassi et al. 2012; Piirsalu et al. 2020; Vichaya et al. 2020; Lasselin et al. 2020). These behavioral features form a major component of the “sick animal behavior” seen in response to LPS, which has been extensively reported in other species. In mouse models, for instance, responses of locomotor activity to LPS and recovery to baseline levels vary, apparently depending on strain/stock, sex, and even the route of LPS administration (Zhao et al. 2017; Meneses et al. 2018). BALB/c and CD-1 mice that received intraperitoneal LPS showed a significant drop in LA with a slower recovery to baseline levels in BALB/c, and in the males compared to females in CD-1 (Meneses et al. 2018). In many other strains of mice, intraperitoneal LPS generally caused a drastic drop in spontaneous locomotor activity (Engeland et al. 2001; Lainiola et al. 2020; Piirsalu et al. 2020). Intracerebroventricular administration to C57BL/6 mice, on the other hand, did not result in any change in LA as analyzed in OFT (Zhao et al. 2017).

In the present study, the NMRs that received LPS injection displayed greatly altered behavior and activity in their home cages, showing dullness, diminished social interactions (diminished tendency to congregate and heap upon one another), decline in movement within the cage, and incoordination. In the OFT, however, the locomotor activity of LPS-treated NMRs, as measured by the number of line crossings, was not significantly reduced compared to control animals. This is despite the doses administered, which we considered to be very high. This observation is different from what has generally been reported in other rodent models where LPS caused a drastic fall in locomotor activity (Engeland et al. 2001; Lainiola et al. 2020; Piirsalu et al. 2020). Studies have shown that reduced motor activity due to LPS or inflammation is mediated by proinflammatory cytokines which induce a reduction of dopamine in basal ganglia that in turn leads to reduced motor activity and motivation (Felger and Miller 2012; Harrison et al. 2015; Felger and Treadway 2017). The depressed motor activity consequent to inflammation is thought to be a mechanism that helps an animal conserve energy which is redirected from growth and reproduction programs into maintenance and survival programs of the body (Wang et al. 2019; Piirsalu et al. 2020). Although LPS caused classical “sick animal behavior” responses in NMR, it failed to depress locomotor activity in the test applied in our study. This could be due to either some unusual interactions between immunity and the brain in the NMR or an extreme tolerance of motor functions or their control centers to the depressive signals from the immune system. This mechanistic aspect was however not assessed in our study, but the finding per se adds a further characteristic of the unique biology of this animal.

TAK 242 is a synthetic cyclohexane derivative that specifically inhibits TLR4 through binding to the intracellular domain (Yamada et al. 2005; Ii et al. 2006; Kawamoto et al. 2008; Ono et al. 2020). This molecule has been shown to protect against inflammatory conditions and various organ injuries (Fenhammar et al. 2011; Zhang et al. 2015; Bhattacharyya et al. 2018; Samarpita et al. 2020). When co-administered with an antibiotic in *Escherichia coli*-induced mouse sepsis model, the agent inhibited increases in serum cytokine levels and improved survival (Takashima et al. 2009). In a clinical trial, however, it failed to suppress cytokine levels in patients with sepsis, or respiratory failure (Rice et al. 2010). A recent study has also shown that pre-treatment with TAK 242 prevents myotube atrophy and muscle wasting caused by LPS (Ono et al. 2020). Although bodyweight loss in NMR injected with LPS 5 mg/kg bwt was significantly lower in the animals that received TAK 242 pre-treatment after 24 h, the overall weight loss due to LPS was not ameliorated by TAK 242 pre-treatment. This is generally similar to what has been reported in several other models (Fellner et al. 2017).

NMRs displayed other unique features of motor activity compared to conventional laboratory rodents. In the open field, away from any support, these animals did not lift their forelimbs off the ground/floor to stand on hind limbs, a feature referred to as rearing. However, the animals did stand on rear limbs with the forepaws placed against the cage wall, a feature referred to as “walling” (Mesembe et al. 2012). This could be due to the design of the locomotor apparatus of this animal that doesn’t allow lifting of the forelimbs without support. The number of wallings was significantly reduced in NMRS that received LPS 5 mg/kg bwt as compared to control but the differences between the other groups were not significant. The animals that received the highest dose of LPS probably avoided walling due to unstable gait which was more pronounced in animals given this dose as indicated by falls in the OFT chamber. Additionally, LPS causes generalized inflammation and intraperitoneal administration could possibly cause peritonitis and inflammation in the abdominal viscera. Lifting the forelimbs and body to lean against the cage wall would increase pressure in the abdomen, which likely elicits pain in inflamed organs, and this could explain reduced walling attempts. The naked mole rat exhibits both slow and fast backward movements (Lacey et al. 2017). In our study, these ‘high-speed’ backward movements were also observed, which, in the home cages, appeared to be elicited by an encounter with an obstacle or ‘unfamiliar’ object. This was also observed in the OFT and it did not appear to be affected by the various treatments. This could be a unique trait of this animal that offers it a quick escape from potential threats in the burrows/tunnels that constitute its natural habitat.

Upon placement and along with the duration of activity recording in the OFT observation chamber, episodes of ‘freezing’ the activity, which is known to occur in other rodent species (Trofimov et al. 2017), were very rare in all groups of NMR. Defecation and urination were also very rare and not affected by the treatments. This points to either a very low anxiety level of this animal when placed in a new environment and the same is not altered by the various agents which were administered, or that anxiety is expressed differently in the NMR.

## Conclusion

LPS caused lethargy, inappetence, loss of body weight, and unstable gait. The loss of weight due to LPS was not significantly ameliorated by TAK 242. Although LA was depressed in home cages after LPS administration, the same was not observed in the OFT chamber nor were the behaviors associated with anxiety such as increased defecation, urination, and episodes of locomotion freezes. This points to a possible exceptional interaction between the brain and immunity. NMR also exhibited some unique behavioral and locomotor features that are not displayed by conventional laboratory rodents in the OFT including the absence of rearing, probably due to the unique design of its locomotor apparatus and high-speed backward movement which could be a mechanism for rapid escape from threats in burrows that constitute its natural habitat. Our results provide further information regarding the unique biology of the NMR particularly pointing to a possibly unusual interaction between immunity and the nervous system. Although additional mechanistic studies are needed to shed more light on this aspect, our findings suggest that the NMR holds great potential as a natural model for studying inflammatory or immune-related diseases.

## Supplementary Information

Below is the link to the electronic supplementary material.

Supplementary file1 A video clip showing backward movements exhibited by NMR in the observation chamber (MP4 4226 kb)

## Data Availability

All data on which the conclusions of the manuscript rely are presented in the main paper or additional supporting files.
